# Surgical management of stage 3 and 4 pressure injuries in trauma patients using ovine forestomach matrix grafts: a prospective case series

**DOI:** 10.3389/fsurg.2026.1704665

**Published:** 2026-04-20

**Authors:** Sophia M. Trinh, Kaitlyn Andre, Ada I. Özcan, Dhanushka S. Vitharana, Paige E. Deville, Joseph W. Mason, John P. Hunt, Alan B. Marr, Patrick P. Greiffenstein, Lance E. Stuke, Alison A. Smith

**Affiliations:** Department of Surgery, Louisiana State University Health Sciences Center, New Orleans, LA, United States

**Keywords:** ovine forestomach matrix, pressure injury, surgical reconstruction, trauma, wound healing

## Abstract

**Introduction:**

Stage 3 and 4 pressure injuries (PIs) pose significant challenges in trauma patients. Surgical management aims to support improvements in tissue vitality and often relies on debridement and negative pressure wound therapy. The use of ovine forestomach matrix (OFM)-based grafts to augment existing surgical approaches may improve tissue quality prior to reconstruction or closure by secondary intention.

**Methods:**

This prospective observational study is part of a larger Institutional Review Board-approved study (Registry: ClinicalTrials.gov. Clinical trial number: NCT05243966). The study enrolled patients with Stage 3 and 4 PIs between July 2022 and July 2024 at a single level 1 trauma center. The study’s primary endpoint was the incidence of postoperative complications and secondary endpoints included time to granulation tissue coverage and/or fill, percent area reduction, and number of OFM applications.

**Results:**

Nine participants (eight men, one woman) with a total of 12 PIs (25% Stage 3 and 75% Stage 4) were enrolled in the study. The mean surface area was 46 ± 24 cm^2^, and 10 of the 12 enrolled PIs included areas of tunneling and/or undermining. The median time to 50% granulation tissue was 2.0 (IQR: 1.5, 8.5) weeks and the median time to complete granulation tissue coverage was 6.5 (IQR: 2.0, 15.0) weeks. Tunneling or undermining was eradicated in 50% of PIs. The mean percent area reduction at the last recorded visit was 61% ± 30%. There were no postoperative complications.

**Conclusion:**

These results suggest that OFM-based grafts may serve as a valuable adjunct for the surgical management of late-stage PIs that are clinically challenging to heal.

## 1 Introduction

Pressure injuries (PIs)—defined as localized damage to the skin and underlying tissue due to prolonged pressure—remain a significant challenge in hospitalized patients and are a common yet often preventable cause of morbidity (1). In 2023 alone, approximately 2.5 million PIs were reported, predominantly in acute care settings, with an estimated cost of $26.8 billion (2). The burden on both the patient and the healthcare system is substantial, as a single PI can generate up to $70,000 in medical expenses over the course of a lengthy recovery, with the majority of costs incurred by the more severe, late-stage pressure injuries (3). Given their high morbidity, these wounds often require prolonged management, including frequent dressing changes, infection control, and surgical intervention. While complete wound closure is the ideal outcome, many patients present with complex PIs where definitive closure may not be feasible due to underlying comorbidities, extensive soft-tissue damage, or limited access to specialized reconstructive surgery. In these cases, meaningful clinical improvement—such as wound size reduction, decreased exudate, granulation tissue formation, and resolution of tunneling or undermining—can significantly alleviate the burden on both patients and caregivers. While optimization of nutritional, physiological, and wound-care management is essential, it will not always result in wound healing, particularly in patients with spinal cord injury with resultant permanent limitations in mobility, vascularity, and tissue quality. As such, adjunctive treatment modalities that can improve tissue quality and promote tissue coverage remain clinically valuable.

Surgical debridement remains a cornerstone of PI management, particularly in cases complicated by necrosis, infection, or non-viable tissue that impairs healing (4). However, debridement alone may not address the underlying tissue deficit, and conventional strategies often rely on prolonged negative pressure wound therapy (NPWT) or traditional wound care to promote granulation tissue formation. In recent years, the integration of bioscaffolds has emerged as a promising adjunct to traditional wound care, offering a means to accelerate tissue regeneration and stabilize wound beds in preparation for either definitive closure or long-term conservative management. For example, Awad et al. proposed an algorithm for the surgical management of Stage 3 and 4 PIs, incorporating both reconstructive and non-reconstructive pathways and utilizing available technologies, including tissue bioscaffolds and NPWT (5).

At our level 1 trauma center, we often encounter late-stage PIs requiring surgical management, which we have typically managed through surgical debridement, patient optimization, and NPWT, with the goal of stabilizing the wound bed and, where possible, filling these typically deep defects with well-vascularized tissue. Recently, we incorporated ovine forestomach matrix (OFM)-based grafts into our surgical protocols for management of PIs. OFM-based grafts have demonstrated potential in managing complex wounds by providing immediate coverage, promoting neovascularization, and supporting granulation tissue development (6–8). The following prospective case series presents our initial findings using OFM-based grafts to augment our surgical protocols to manage these complex soft-tissue defects.

## 2 Materials and methods

### 2.1 Ethics statement

This study was conducted in accordance with the principles of the Declaration of Helsinki. Approval was granted by a central Institutional Review Board (Advarra, Maryland, USA) (NCT05243966). Written informed consent was obtained from all participants included in the study.

### 2.2 Study design and setting

All participants who were otherwise undergoing reconstruction with OFM grafts were eligible for enrollment. This preliminary report is a single arm analysis of those patients that had PIs reconstructed with OFM grafts between July 2022 and July 2024 at a single center. All PIs were surgically debrided under general anesthesia to remove necrotic and/or infected tissue, as needed. Following debridement, photographs were taken and dimensions of the defects were recorded. OFM—either in the graft form (Myriad Matrix™ Soft Tissue Bioscaffold, Aroa Biosurgery Limited, Auckland, New Zealand) or in the granular format (particulate or powder form) (Myriad Morcells™, Aroa Biosurgery Limited, Auckland New Zealand)—was placed in the wound bed in accordance with the manufacturer's instructions. The choice of graft versus granular format was made at the discretion of the operating surgeon. The grafts were covered with a non-adherent primary dressing (Xeroform™, McKesson Medical-Surgical, Richmond, VA, USA) and a secondary dressing, with either gauze or NPWT, depending on the surgeon's clinical judgement. Dressings were monitored and maintained for at least 7–10 days before removal and evaluation of the defect. Defects were dressed appropriately by the study team every 5–7 days during the postoperative follow-up period.

### 2.3 Data collection

Patient demographics, baseline PI characteristics, outcomes and postoperative complications were recorded prospectively. Given this is a cohort from a prospective study, the primary study outcome was postoperative complications and device-related adverse events to ensure safety assessment. Secondary endpoints captured clinically meaningful measures of wound progression and were the time to granulation coverage and/or fill, percent area reduction, and number of OFM applications.

### 2.4 Statistical analysis

Descriptive statistics were calculated using GraphPad Prism (version 10.1.2, GraphPad Software, LLC, Boston, MA, USA). The normality of distribution of continuous variables was tested using the Shapiro–Wilk test. Continuous variables with normal distribution were presented as mean ± SD; non-normal variables were reported as median (IQR, interquartile range for Q1 and Q3).

## 3 Results

To date, nine participants have been recruited to the study (Table 1), all of whom presented to our trauma center requiring surgical intervention for a PI. Participants included eight (89%) men and one (11%) woman, with six having a history of spinal cord injury. Mean patient age and BMI were 45.9 ± 19.1 years and 24.0 ± 6.8, respectively. Two patients (22%) were current smokers, and none had diabetes or vascular disease. Two participants had more than one PI, with a median of 1.0 (IQR: 1.0, 1.5) PIs per patient across the entire cohort, accounting for a total of 12 PIs enrolled. The median length of stay following application of the OFM grafts was 7.0 (IQR: 3.0, 37.5) days.

**Table 1:** Patient demographics.

Participant ID	Gender	Age	BMI	SCI	Smoking status	Defects per patient	LOS
1	Male	22	18.5	None	No	1	37
2	Male	50	36.8	Paraplegic	No	1	7
3	Male	36	21.2	None	Yes	1	60
4	Male	34	21.4	Paraplegic	No	2	2
5	Male	35	22.6	Paraplegic	Yes	3	5
6	Male	54	25.8	Paraplegic	No	1	4
7	Female	33	14.7	None	No	1	26
8	Male	82	22.8	Paraplegic	No	1	15
9	Male	67	32.5	Paraplegic	No	1	2
Mean ± SD	–	45.9 ± 19.1	24.0 ± 6.8	–	–	–	–
Median [IQR]	–	–	–	–	–	1.0 [1.0, 1.5]	7.0 [3.0, 37.5]

SCI, spinal cord injury; LOS, length of stay; SD, standard deviation; BMI, body mass index; IQR, interquartile range.

**Figure 1:**
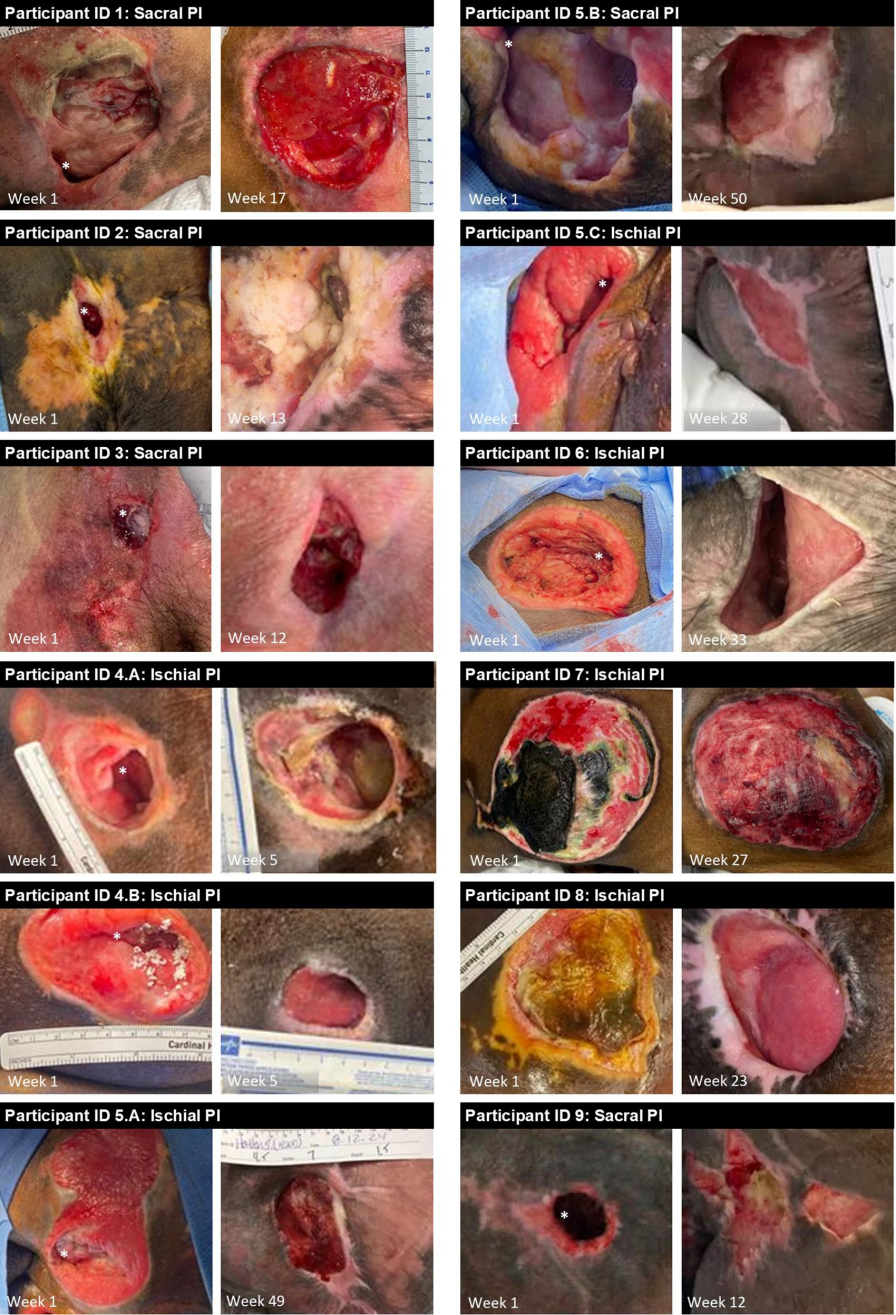
Representative images of pressure injuries at intraoperative placement of OFM grafts (“Week 1”), and at last recorded follow-up visit. Pressure injury IDs correspond to participant IDs from Tables 2 and 3 respectively.

Among the 12 PIs were three stage 3 (25%) and nine stage 4 (75%) PIs, with a mean surface area of 46 ± 24 cm^2^ (Table 2). Eight of the PIs were greater than 1 month old, and one participant presented with two stage 4 PIs estimated to be 2–5 years old. Two PIs (17%) had confirmed osteomyelitis, and 10 PIs (83%) had undermining or tunneling at presentation. The median product application was 1.0 (IQR: 1.0, 1.8) across the entire cohort, with only three PIs requiring a second application of OFM grafts (Table 2). Four PIs (33%) were managed concomitantly with NPWT. The mean follow-up period across the cohort was 23 ± 15 weeks, with a range of 5–50 weeks. Intraoperative images of the PIs at the time of OFM graft application and at the last recorded study visit are provided in Figure 1. The median time to 50% coverage and/or fill of the defects was 2.0 (IQR: 1.5, 8.5; *n* = 9 defects) weeks across the nine defects that could be assessed. Eight defects (67%) were monitored until complete granulation tissue coverage and/or fill, with the remaining defects being lost to follow-up prior to this assessment. From these defects, the median time to tissue coverage was 6.5 (IQR: 2.0, 15.0; *n* = 8 defects) weeks. Of the 10 PIs with undermining or tunneling, six (67%) had eradicated these voids at the last follow-up. In the remaining four, the areas of undermining or tunneling were reduced at the last follow-up visit. While none of the PIs fully epithelialized by the last follow-up visit, the mean PAR at the last recorded visit was 61 ± 30%. There were no immediate postoperative complications (i.e., infections, hematomas, graft loss) reported across the cohort.

**Table 2:** Baseline PI and postoperative outcomes.

PI ID	Stage	Location	PI age	Area (cm^2^)	Tunneling/undermining	Osteomyelitis	Number of product applications	NPWT	Length of follow-up (weeks)	Time to 50% granulation tissue (weeks)	Time to complete granulation tissue (weeks)	Percent area reduction	Tunneling/undermining eradicated
1	4	Sacral	1–6 m	29	Yes	Yes	2	Yes	17	5	12	−7	Resolved
2	4	Sacral	6–12 m	15	Yes	Yes	1	No	13	1	LTFU	65	Improved
3	4	Sacral	1–6 m	32	Yes	No	1	No	12	12	LTFU	94	Resolved
4A	4	Ischial	2–5 y	32	Yes	No	1	No	5	1	LTFU	38	Improved
4B	4	Ischial	2–5 y	30	Yes	No	1	No	5	ND	1	90	Resolved
5A	3	Ischial	1–6 m	75	Yes	No	1	No	49	13	27	65	Resolved
5B	3	Sacral	1–6 m	30	Yes	No	1	No	50	2	LTFU	53	Improved
5C	3	Ischial	1–6 m	67	Yes	No	1	No	28	ND	2	85	Resolved
6	4	Ischial	1–6 m	70	Yes	No	2	Yes	33	2	16	71	Improved
7	4	Ischial	1–6 m	91	No	No	2	Yes	29	5	9	22	NA
8	4	Ischial	1–6 m	38	No	No	1	No	28	2	4	66	NA
9	4	Sacral	<1 m	50	Yes	No	1	Yes	12	ND	2	94	Resolved
Mean ± SD	–		–	46 ± 24	–	–	–	–	23 ± 15	–	–	61 ± 31	–
Median [IQR] (*n*)	–		–	–	–	–	1.0 (1.0, 1.8)	–	–	2.0 [1.5, 8.5] (9)	6.5 [2.0, 15.0] (8)	–	–

PI, pressure injury; SD, standard deviation; IQR, interquartile range; m, month; y, year; NPWT, negative pressure wound therapy; n, sample size; ND, not determined; LTFU, lost to follow-up.

### 3.1 Case example 1

**Figure 2:**
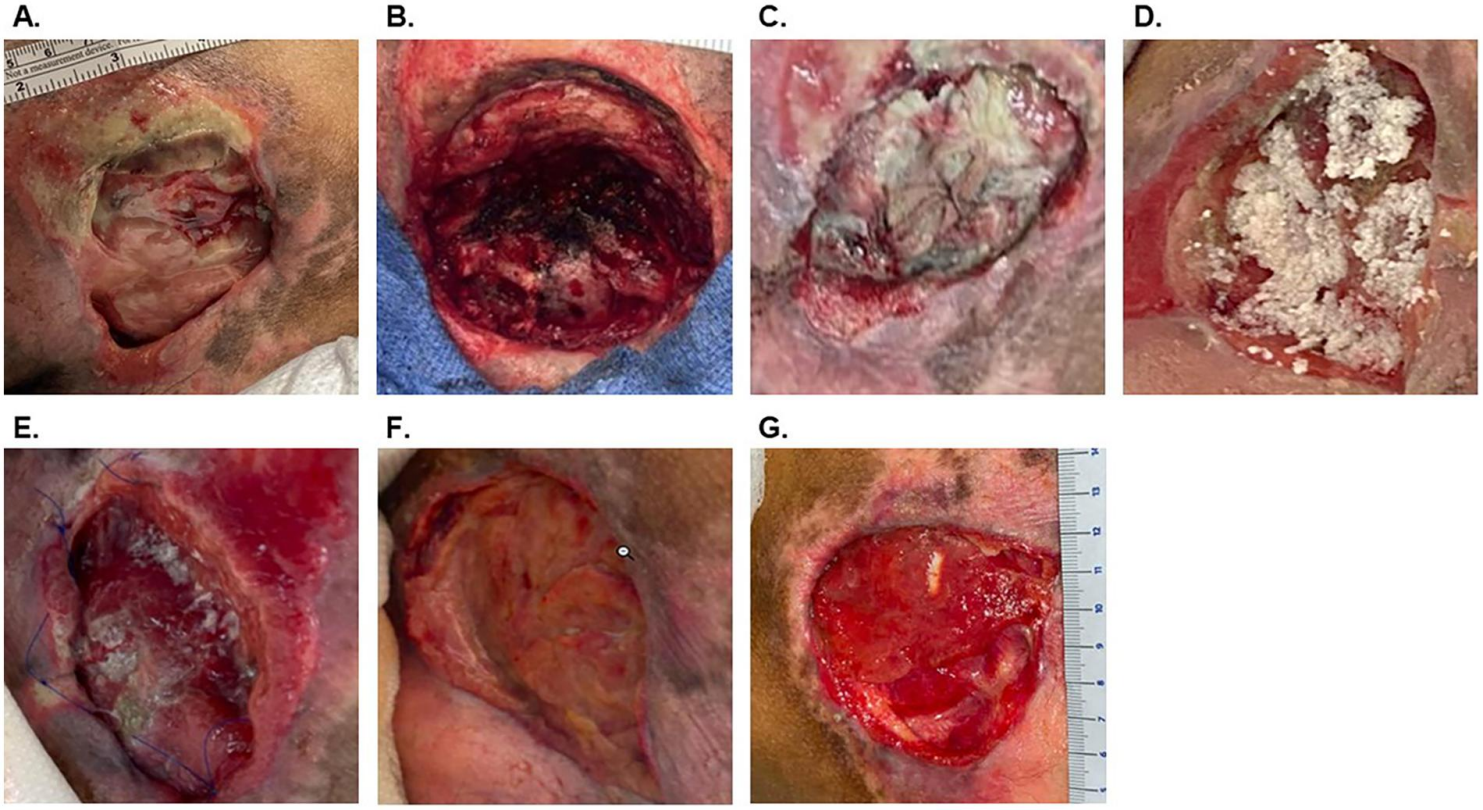
Case Example 1, Participant ID 1. **(A)** Initial sacral wound prior to debridement. **(B)** Wound following debridement (29 cm^2^). **(C)** Postoperative day 10. **(D)** Four weeks after the index procedure and following a second application of OFM particulate. **(E)** Five weeks after the index procedure. **(F)** Twelve weeks after index procedure. **(G)** Sixteen weeks after initial OFM placement (31 cm^2^).

A 22-year-old man (participant ID: 1, Tables 1, 2) presented with a chronic sacral PI with associated osteomyelitis. Initially, the wound was treated with local wound care approaches, and the patient received multiple rounds of antibiotic therapies for osteomyelitis before surgical intervention was pursued (Figure 2A). The patient was taken to the operating room where the wound was sharply debrided until only healthy tissue remained, leaving a defect of approximately 29 cm^2^ with substantial depth (Figure 2B). OFM particulate (1,000 mg) was placed in the debrided defect, followed by an OFM graft (7 × 10 cm, three-layer) to cover the particulate and the remaining wound bed. The OFM was trimmed to meet the edges of the wound, covered with a non-adherent dressing, and then both were secured in place with polyglycolic acid (PGA) sutures to ensure that the OFM graft remained undisturbed. Standard NPWT (125 mmHg) was applied as a secondary dressing and removed on postoperative day 10. At the first dressing change (Figure 2C), unincorporated OFM graft was visible on the surface of well-vascularized granulation tissue that had begun to fill the defect. At 4 weeks following the index procedure, there was noticeable volumetric fill of the defect; however, a second application of OFM particulate (100 mg) was undertaken to further bring the base of the defect to the level of the surrounding tissue (Figure 2D). Sixteen weeks after the index procedure, the defect was approximately 31 cm^2^. While there was a slight increase in wound size measurement, the defect demonstrated a uniformly healthy-appearing wound bed, comprising granulation tissue to the level of the surrounding skin. Areas of tunneling on initial presentation had been eradicated. The patient did not require any additional debridement.

### 3.2 Case example 2

**Figure 3:**
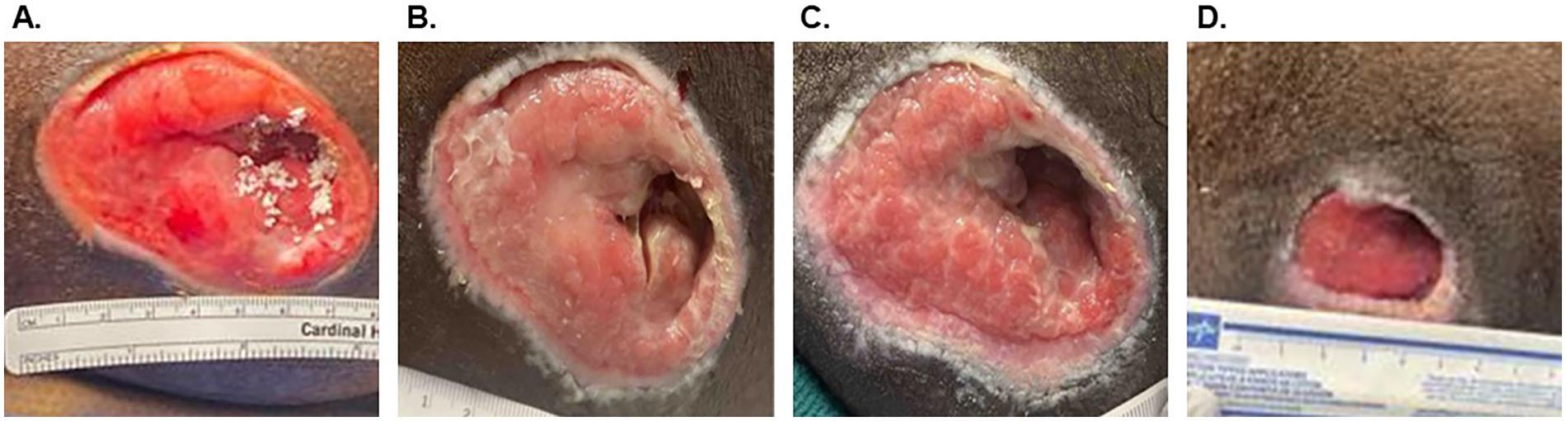
Case Example 2, Participant ID 4. **(A)** Initial ischial pressure injury (30 cm^2^) showing placement of the OFM particulate, prior to OFM graft placement. **(B)** One week after OFM placement. **(C)** Two weeks after the index procedure. **(D)** Five weeks after OFM placement (3 cm^2^).

A 34-year-old man (participant ID: 4, Table 1) presented to the hospital with spinal cord injury from a motor vehicle accident, resulting in paraplegia. The patient subsequently developed chronic ischial PIs over the course of his inpatient stay. Initial management with off-loading and standard wound care failed to resolve the PIs, and the patient was referred to the trauma team for surgical intervention. The left ischial PI (Table 2, PI ID: 4B), measuring 30 cm^2^, was found to be clean and viable and therefore did not require surgical debridement (Figure 3A). Particulate OFM (500 mg) and OFM graft (10 × 10 cm, three-layer) were applied over the wound bed. The OFM particulate was placed primarily into an area of deep tunneling (Figure 3A). The OFM graft was trimmed to match the size of the wound, and a non-adherent primary dressing was secured in place with staples. A secondary dressing was applied using gauze and foam dressing materials. The staples and secondary dressing were removed 7 days after placement, revealing a healthy-appearing wound base. The wound area progressed to 3 cm^2^ (90% PAR) by the fifth week, with nearly complete resolution of the deep tunneling. The patient required no additional debridements during the 5 weeks and there were no postoperative complications noted.

### 3.3 Case example 3

**Figure 4:**
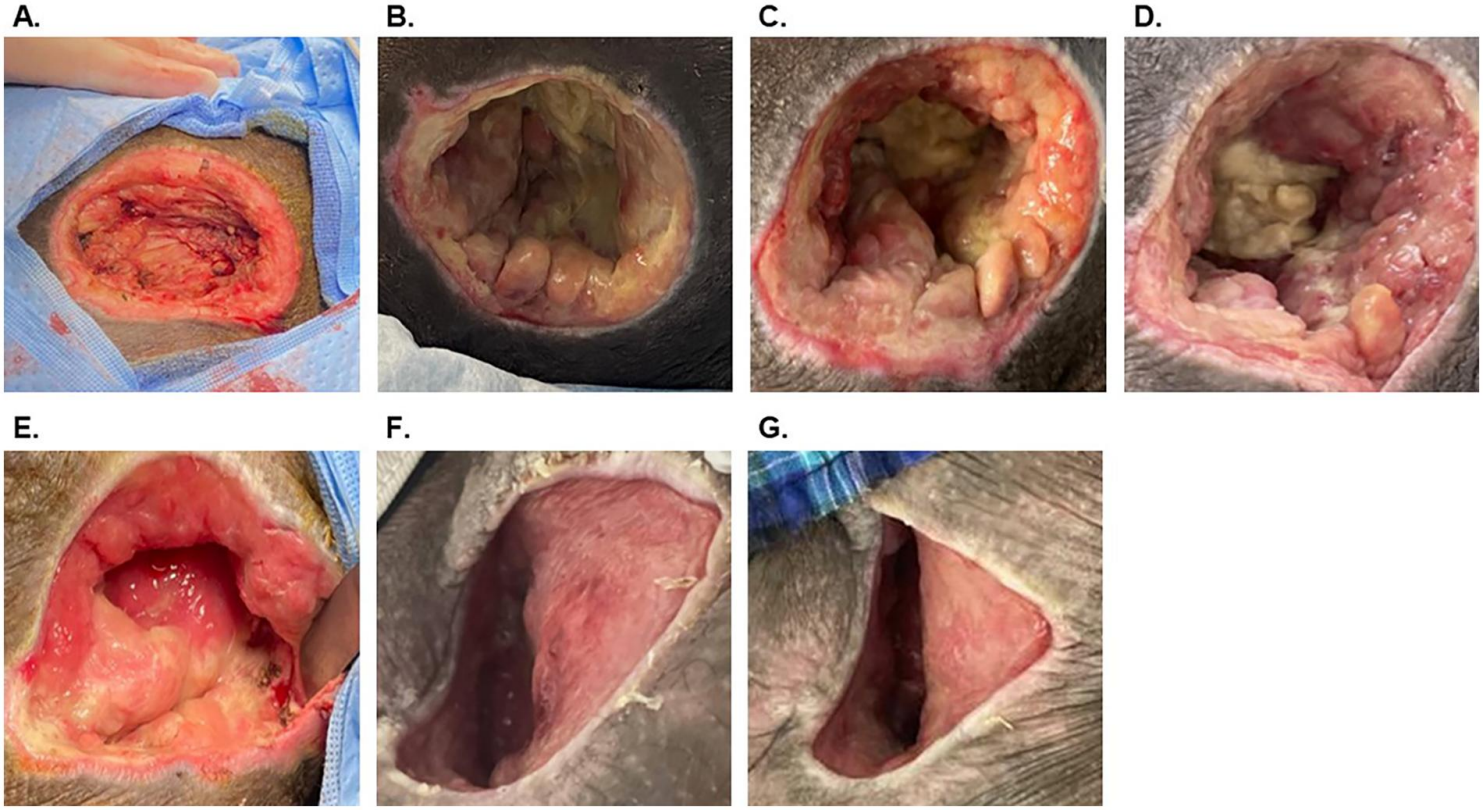
Case Example 3, Participant ID 6. **(A)** Initial ischial pressure injury after debridement (70 cm^2^). **(B)** Approximately 2 weeks after OFM placement. **(C)** Four weeks after OFM placement. **(D)** Six weeks after OFM placement. **(E)** Twelve weeks after OFM placement, repeat debridement with additional OFM sheet and particulate placement (70 cm^2^). **(F)** Twenty-four weeks after initial OFM placement. **(G)** Thirty-two weeks after initial OFM placement (20 cm^2^).

A 54-year-old man (participant ID: 6, Tables 1, 2) presented to the hospital with spinal cord injury and paraplegia due to a gunshot wound. During his hospital stay, the patient developed a chronic sacral PI and was referred for surgical debridement and reconstruction (Figure 4A). The wound measured 70 cm^2^ after debridement, and OFM particulate (500 mg) and an OFM graft (10 × 10 cm, three-layer) were applied over the remaining wound bed with a non-adherent primary dressing secured in place with staples. A secondary dressing was applied using gauze and foam dressing materials. At dressing changes 2 and 6 weeks following application, the wound showed intermittent progress with graft integration and the development of healthy granulation tissue. The patient was then lost to follow-up until readmission for a soft-tissue infection of the scrotum 12 weeks after initial OFM application. The sacral PI was found to contain necrotic tissue with active fibrinous exudate production, requiring additional surgical debridement. The remaining wound bed measured 70 cm^2^ following debridement in the operating room, although it had demonstrated depth reduction since the initial application (Figure 4E). A second application of OFM particulate (500 mg) and an OFM graft (10 × 10 cm, three-layer) was performed, dressed as previously described, with NPWT (125 mmHg) placed as the secondary dressing. Thirty-two weeks after the initial procedure, the defect measured 20 cm^2^ and was observed to have a healthy-appearing bed with granulation tissue.

## 4 Discussion

PIs remain a significant challenge in clinical practice, particularly among patients with spinal cord injuries or other conditions that impair mobility and increase the risk of tissue damage. This patient population often faces additional barriers to effective wound management, including socioeconomic constraints that limit access to follow-up care and interdisciplinary wound treatment (9, 10). This is especially true in the trauma and acute care surgery (ACS) setting, where patients with PIs may be transitory. In such cases, the primary aim is to stabilize the patient prior to discharge to facilities better resourced for long-term care. Prior work has highlighted persistent barriers to effective pressure injury management in acute and high-acuity settings, including inadequate clinician knowledge, time constraints, limited patient participation due to pain or cognitive impairment, and practical challenges associated with implementing evidence-based protocols in trauma and ACS environments (11). This makes long-term management of the PIs within the trauma and ACS setting often unfeasible. In addition, general, trauma, and acute care surgeons in these environments often have limited bandwidth for specialized, on-going wound management of PIs. A subset of late-stage PIs may be suitable for reconstructive closure (12). However, given the complex nature of flap-based reconstruction, these approaches typically fall under the domain of reconstructive and plastic surgery (13), a specialization that may not be available to PIs in the trauma and ACS setting.

In our level 1 trauma center, we are often consulted or referred for non-reconstructive surgical management of PIs. Our immediate surgical goals are not necessarily to close the wound, but rather to use existing tools like surgical debridement, NPWT, off-loading, and nutritional optimization to stabilize the PI and improve the overall tissue quality of the wound bed. If the PI can be stabilized and tissue quality improved, downstream reconstructive procedures or closure via secondary intention may become feasible (4, 14). Moreover, any improvements to the wound bed—for example, tissue coverage of exposed structures or tissue fill of volumetric loss, undermining and tunneling—significantly reduce the complexity of managing these wounds with traditional wound care. Thorough debridement of the PI is the mainstay of our non-reconstructive surgical management of these wounds. Debridement to address bacterial contamination, biofilm, and necrotic tissue is a necessity to achieve optimal outcomes (12). Given the growing body of evidence supporting the use of OFM-based grafts to restore well-vascularized tissue coverage and provide tissue fill for deep defects (6, 7, 15), we considered augmenting our typical surgical debridement and NPWT protocols with OFM grafts in order to expedite stabilization of PIs and improve the quality of tissue within the wound bed.

The primary objectives of our approach with OFM-based grafts were to provide immediate coverage of the wound bed and reduce the complexity of wound management by addressing depth, undermining, and tunneling. In this way, we were better able to manage these wounds with traditional wound care and could potentially set these PIs up for future reconstructive surgical management. Across the 12 PIs reported herein, we saw meaningful improvements in all PIs. For example, the quality of the vascularized tissue had markedly improved across the cohort with noticeable tissue infill, coverage, and eradication or reduction of tunneling/undermining (Table 1; Figure 1). The primary objective of our surgical intervention was not ultimate wound closure; however, we observed wound area reduction in all except one of the PIs managed with OFM-based grafts (Table 1). Despite not realizing significant wound reduction while in our care, this patient did show substantial progress with eradiation of tunneling and achieved a wound state supportive of definitive closure.

PIs are prone to complications due to the poor quality of the underlying tissue, microbial contamination, and patient factors. For example, Lefevre et al. (16) reported a 34.5% complication rate—including infection, delayed healing, and necrosis—following reconstructive closure of PIs. Similarly, Biglari et al. (17) reported a 21% complication rate—including dehiscence and partial flap necrosis—following PI reconstruction. Even in cases where PIs were managed by traditional wound care, complication rates, particularly infection rates, remain high (18). In our current cohort, there were no immediate postoperative complications (e.g., infection, graft loss), underscoring not only the safety of OFM-based grafts but also the tolerance of these grafts to bacterial contamination. A recently published preclinical study highlighted the stability of OFM and its resistance to enzymatic degradation compared to other bioscaffolds (19). A clinical publication of 130 lower extremity defects that received OFM further reaffirms these findings, which reported a median of one OFM application and no instances of graft infection or graft loss despite 93.8% of defects being reported as CDC Grade 3 and 47.8% being positive for osteomyelitis (20). Similarly, a recent prospective analysis of 49 patients managed with OFM at Level 1 trauma centers showed no graft loss and a median of 1 OFM application (21).

There are numerous tissue-derived bioscaffolds now available for wound care and reconstructive applications, and several have been studied in relation to PI management and treatment (22–24). Typically, these types of technologies are applied on a weekly basis, either in the operating room at the time of debridement or in the outpatient wound care setting. For example, Kim et al. demonstrated that application of acellular dermal matrix graft improved closure rates in hard-to-heal wounds including PIs, but required three product applications (24). We have found that OFM-based grafts are typically only applied once; for example, across the current cohort, the median product application was 1.0 (IQR: 1.0, 1.8) (Table 2). This result is in line with other clinical studies using OFM-based grafts in chronic and contaminated soft-tissue defects (8, 25, 26). The persistent nature of these grafts in these hostile environments, including PIs, may be attributed to the anti-inflammatory components present in the material that slow the proteolytic degradation of OFM within a chronic wound bed (27). This practical observation has important implications for PI patients, who often face significant risks with repeated surgeries or prolonged recovery times, making a one-time application an important benefit. For example, a nationwide population-based cohort study found that patients with preoperative PIs had increased risks of postoperative complications—including septicemia, pneumonia, and stroke—as well as a significantly higher 30-day postoperative mortality rate (28). Therefore, managing these late-stage PIs with OFM grafts, compared to alternate tissue-derived bioscaffolds, potentially minimizes patient exposure to the operating room and therefore reduces operative risks for these patients.

Based on our positive outcomes to date, the integration of OFM as an adjunct to surgical debridement of PIs in trauma and ACS settings may bridge the gap between wound stabilization and eventual reconstruction. By fostering vascularized tissue development and facilitating wound healing with a single application, OFM provides a valuable tool for surgeons who may lack reconstructive expertise but still need to surgically manage complex PIs effectively. This approach allows acute care teams to focus on their primary responsibilities while ensuring that PI patients receive effective surgical intervention without requiring specialist surgical expertise. Moreover, the use of OFM-based grafts may enhance patient flow by enabling faster transition from trauma and ACS services, reducing hospital length of stay, and minimizing the need for specialized postoperative care.

### 4.1 Limitations

As a small case series (*n* = 9 patients, 12 PIs), this study was not without limitation and the results should be interpreted appropriately. Notably, the small sample size and absence of a comparator cohort limits the generalizability of our findings, and also limited our ability to conduct subgroup analysis, including comparisons between wounds treated with and without NPWT or with different dressing change algorithms. A larger cohort with longer follow-up would be necessary to confirm the long-term efficacy of OFM-based grafts in PI management and to make comparative claims regarding potential additive effects of NPWT and OFM or impact of wound location or chronicity. While OFM demonstrated a low complication rate, direct comparisons with other treatment modalities in a randomized controlled trial setting would be beneficial to further establish its comparative effectiveness. Finally, our study focused primarily on short-term outcomes. Further research is needed to assess the durability of PIs healed with OFM and its potential role in facilitating later reconstructive procedures.

## 5 Conclusions

In this prospective case series, OFM proved to be a successful tool in the management of late-stage PIs within our level 1 trauma center. Rather than relying solely on debridement and NPWT, which primarily offer stabilization and protection of the wound bed, incorporation of OFM grafts enabled a more proactive approach geared toward improving the quality of the wound bed. Across the current cohort, we augmented our existing surgical debridement protocols with OFM-based grafts to provide more immediate tissue coverage, reduced complications, and simplified wound management. Given the increasing incidence of PIs in trauma and ACS settings and the limited availability of specialized reconstructive expertise, OFM offers a practical and effective addition to the surgical armamentarium for the treatment of these high-risk patients.

## Data Availability

Requests to access the datasets should be directed to the corresponding author.
